# Trimethoprim-Sulfamethoxazole (Bactrim) Dose Optimization in *Pneumocystis jirovecii* Pneumonia (PCP) Management: A Systematic Review

**DOI:** 10.3390/ijerph19052833

**Published:** 2022-02-28

**Authors:** Abdul Haseeb, Mohammed A. S. Abourehab, Wesam Abdulghani Almalki, Abdulrahman Mohammed Almontashri, Sultan Ahmed Bajawi, Anas Mohammed Aljoaid, Bahni Mohammed Alsahabi, Manal Algethamy, Abdullmoin AlQarni, Muhammad Shahid Iqbal, Alaa Mutlaq, Saleh Alghamdi, Mahmoud E. Elrggal, Zikria Saleem, Rozan Mohammad Radwan, Ahmad Jamal Mahrous, Hani Saleh Faidah

**Affiliations:** 1 Department of Clinical Pharmacy, College of Pharmacy, Umm Al-Qura University, Makkah 21955, Saudi Arabia; ph.wesam@outlook.sa (W.A.A.); xabdullrahman22@gmail.com (A.M.A.); sultanahmed199717@gmail.com (S.A.B.); merggal@uqu.edu.sa (M.E.E.); ajmahrous@uqu.edu.sa (A.J.M.); 2 Department of Pharmaceutics, Faculty of Pharmacy, Umm Al-Qura University, Makkah 21955, Saudi Arabia; maabourehab@uqu.edu.sa; 3 Department of Internal Medicine, Alnoor Specialist Hospital, Makkah 21955, Saudi Arabia; anas.aljoaid@gmail.com (A.M.A.); b7nitts@gmail.com (B.M.A.); 4 Department of Infection Prevention and Control Program, Alnoor Specialist Hospital Makkah, Makkah 21955, Saudi Arabia; mmalgethamy@moh.gov.sa; 5 Infectious Diseases Department, Alnoor Specialist Hospital Makkah, Makkah 21955, Saudi Arabia; al-qrni@hotmail.com; 6 Department of Clinical Pharmacy, College of Pharmacy, Prince Sattam bin Abdulaziz University, Alkharj 11942, Saudi Arabia; m.javed@psau.edu.sa; 7 General Department of Pharmaceutical Care, Ministry of Health, Riyadh 12211, Saudi Arabia; asmutlaq@moh.gov.sa; 8 Department of Clinical Pharmacy, Faculty of Clinical Pharmacy, Al Baha University, Al Baha 57911, Saudi Arabia; saleh.alghamdi@bu.edu.sa; 9 Department of Pharmacy Practice, Faculty of Pharmacy, The University of Lahore, Lahore 40050, Pakistan; xikria@gmail.com; 10 Pharmaceutical Care Department, Alnoor Specialist Hospital Makkah, Makkah 21955, Saudi Arabia; dr.rozan@hotmail.com; 11 Department of Microbiology, Faculty of Medicine, Umm Al Qura University, Makkah 21955, Saudi Arabia; hsfaidah@uqu.edu.sa

**Keywords:** *Pneumocystis jirovecii* pneumonia, dose optimization, trimethoprim-sulfamethoxazole, co-trimoxazole

## Abstract

(1) Background: *Pneumocystis jirovecii* pneumonia (PCP) has a substantial impact on the morbidity and mortality of patients, especially those with autoimmune disorders, thus requiring optimal dosing strategies of Trimethoprim–Sulfamethoxazole (TMP-SMX). Therefore, to ensure the safety of TMP-SMX, there is a high demand to review current evidence in PCP patients with a focus on dose optimization strategies; (2) Methods: Various databases were searched from January 2000 to December 2021 for articles in English, focusing on the dose optimization of TMP-SMX. The data were collected in a specific form with predefined inclusion and exclusion criteria. The quality of each article was evaluated using a Newcastle–Ottawa Scale (NOS) for retrospective studies, Joanna Briggs Institute (JBI) critical checklist for case reports, and Cochrane bias tool for randomized clinical trials (RCTs); (3) Results: Thirteen studies met the inclusion criteria for final analysis. Of the 13 selected studies, nine were retrospective cohort studies, two case reports, and two randomized controlled trials (RCT). Most of the studies compared the high-dose with low-dose TMP-SMX therapy for PCP. We have found that a low dose of TMP-SMX provides satisfactory outcomes while reducing the mortality rate and PCP-associated adverse events. This strategy reduces the economic burden of illness and enhances patients’ compliance to daily regimen plan; (4) Conclusions: The large-scale RCTs and cohort studies are required to improve dosing strategies to prevent initial occurrence of PCP or to prevent recurrence of PCP in immune compromised patients.

## 1. Introduction

*Pneumocystis carinii* pneumonia (PCP), also known as *Pneumocystis jirovecii* pneumonia has remained the most frequent and highly morbid fungal infection for patients with autoimmune disorders [1,2]. The incidence of PCP was more than 50% in immunocompromised patients, 22–45% in patients with hematological malignancy, 5–15% in transplant recipients and around 2% in patients with rheumatoid diseases [3,4]. The clinical indications of PCP, such as tachycardia, hypoxia, tachypnea, shortness of breath, etc., are the major causes of death of immunosuppressive patients [5]. Therefore, PCP is considered a hallmark disorder indicating human immunodeficiency virus (HIV) infection [5]. In the past few years, the occurrence or recurrence of PCP in HIV-positive patients has been reduced due to advanced technology and tools in diagnostic process, allowing early diagnosis; advanced management strategies in intensive care setting; and advanced preventive measures [6].

Co-trimoxazole is the combination of trimethoprim (TMP), which is a pyrimidine analog, and sulfamethoxazole (SMX), which is from the sulfonamide family [7]. TMP-SMX has been observed to substantially minimize the incidence of PCP in patients. There have been some published data on this topic. A retrospective study reported that the incidence of PCP and PCP-associated mortality was lower in patients with rheumatoid arthritis who received high-dose of glucocorticoids [8]. Another study reported that PCP developed in up to 40% of patients with the lymphoproliferative disease or acute lymphoblastic leukemia, and about 50% of patients experience hepatotoxicity [5]. Moreover, the incidence of adverse events (AEs) was decreased in non-HIV patients who received TMP-SMX prophylactically [9,10].

As with other antibiotics, TMP-SMX must be administered in an appropriate way to achieve adequate antimicrobial activity while reducing concentration-dependent toxicities, necessitating the avoidance of excessive dosage [11]. Specifically, in the era of antimicrobial resistance, optimal antibiotics usage is very crucial to ensure effectiveness of therapy [12]. The TMP-SMX dosing recommendations, on the basis of TMP component, is 320–640 mg/day, administered every 12 h, orally, to treat bacterial infections, and 15–20 mg/kg every 6 to 8 h intravenously then orally for the treatment of PCP infections [7]. In accordance with current dosing guidelines, the recommended therapy for PCP is high-dose TMP-SMX (TMP 15–20 mg/kg/day and SMX 75–100 mg/kg/day for 2–3 weeks) [13]. There is a high incidence of AEs in patients receiving recommended high doses of TMP-SMX. Due to insufficient data on the optimal dose of TMP-SMX, its use is limited. In randomized trials where various treatment regimen was compared for virus-associated PCP, patients receiving recommended high dose of TMP-SMX as a first-line treatment regimen had higher rates of AEs such as skin irritation (e.g., rashes), gastrointestinal disturbances, bone marrow suppression, renal impairment, hepatotoxicity as well as electrolyte disorder for which alternative treatment is required to avoid these AEs [14,15,16,17]. The therapeutic option for treatment of PCP requires a dose of ≥16 mg/kg of TMP-SMX with risk of hepato-renal AEs [18]. Therefore, to ensure the safety of TMP-SMX, there is a high demand to review current evidence in PCP patients with a focus on dose optimization strategies. Dose optimization is the key methods in antimicrobial stewardship programs and very effective to ensure therapeutic outcome of antimicrobial therapy as like in management of PCP [19,20].

## 2. Materials and Methods

### 2.1. Data Sources and Search Strategy

The systematic search assessing the dosing guidelines of TMP-SMX in patients was carried out using Preferred Items for Systematic Reviews and Meta-analysis (PRISMA) guidelines [19,21]. Free-text web searches using Google Scholar, and databases such as PubMed, ScienceDirect, EMBASE, Scopus, the Cochrane Database for Systematic Reviews, etc., were explored for articles in English from January 2000 to December 2021. Reference lists of relevant studies were screened for additional titles for inclusion in the review. The keywords used for the search were “dose optimization”, “*Pneumocystis carinii* pneumonia”, “*Pneumocystis jirovecii* pneumonia”, “HIV-patients”, “immune compromised patients” “Co-trimoxazole”, “trimethoprim/sulfamethoxazole”, and “TMP-SMX”. 

### 2.2. Selection Criteria and Procedure

The studies reporting the dosing strategy of TMP-SMX in the patients for the occurrence or recurrence of PCP were included in this review. Based on the titles and abstracts, studies of all types with any data on clinical outcomes of TMP-SMX regimens were included for further screening. The retrospective studies, randomized controlled trials (RCTs), and case reports available in English were included in this study. The studies published before January 2000 or having inappropriate and incomplete information were excluded from the study. Titles and abstracts of all included articles collected through the search were screened by two reviewers independently. In case of uncertainty as to whether selected studies met inclusion criteria, they discussed with a third reviewer.

### 2.3. Data Extraction

Data extraction was performed using the predesigned data collection form for this review using Microsoft Word 2013. Information retrieved from the selected articles included author and year, design, sample size, characteristics of patients, dosing strategy, clinical outcomes, and findings. Data extraction was performed by one of the reviewers and reviewed by another co-author. Any discrepancies were resolved by a third reviewer.

### 2.4. Article Quality Assessment

The quality of each article was evaluated using a Newcastle–Ottawa Scale (NOS) for retrospective studies, Joanna Briggs Institute, The University of Adelaide (JBI, Adelaide, South Australia) critical checklist for case reports, and Cochrane bias tool for randomized clinical trials (RCTs) [22,23,24]. Two of the reviewers assessed the quality of each included study independently. They compared their results and disagreements were resolved by detailed discussion.

## 3. Results

### 3.1. Study Characteristics

**Figure 1:**
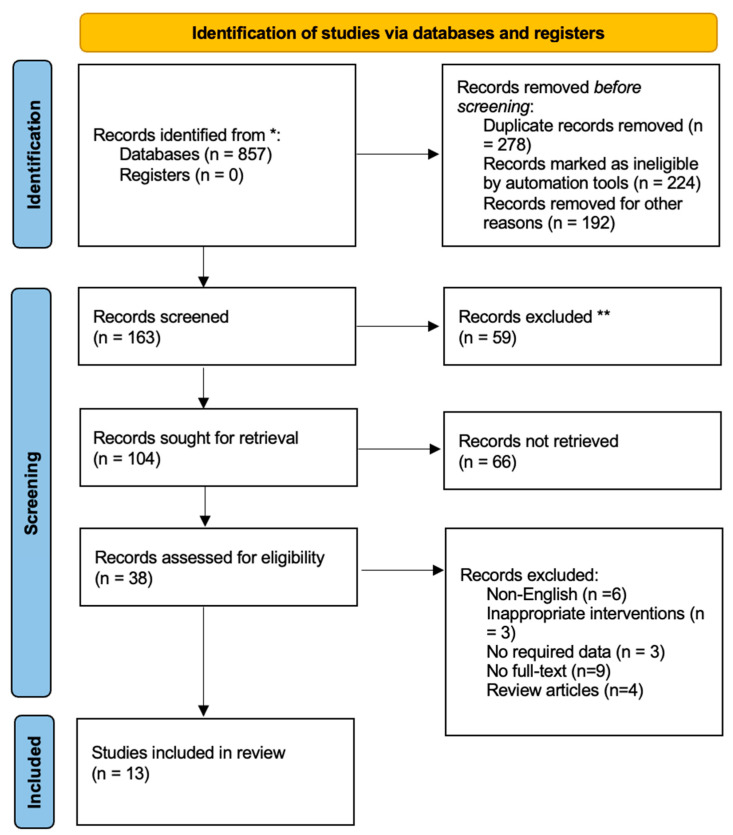
Flow diagram of selection of includes studies. * Consider, if feasible to do so, reporting the number of records identified from each database or register searched (rather than the total number across all databases/registers). ** If automation tools were used, indicate how many records were excluded by a human and how many were excluded by automation tools.

In total, 857 related published studies were identified from grey literature as well as electronic databases. After the removal of the duplicate studies and other reasons, 163 studies were evaluated for eligibility criteria. Based on inclusion and exclusion criteria, 125 studies were excluded after the screening of the titles and abstracts. Most of the studies were identified through reference snowballing. Full-text articles were then screened for final analysis. Twenty-five articles were excluded due to the following reasons: no full-text (*N* = 09), literature reviews (*N* = 04), inappropriate intervention (*N* = 03), no required data (*N* = 03), and non-English (*N* = 06). A total of 13 articles met the inclusion criteria for final analysis [3,8,13,25,26,27,28,29,30,31,32,33,34]. Of the 13 selected studies, nine were retrospective cohort studies [8,13,25,26,27,28,29,30,34], two case reports [31] and two randomized controlled trial (RCT) [3,33]. The PRISMA flow diagram reporting the procedure of selection of studies is shown in Figure 1. 

The studies collected were published after 2000. The main characteristics of selected studies were discussed in Table 1. A total of 2663 patients were recruited in the included studies.

### 3.2. Quality Assessment of Studies

The outcomes of NOS, Cochrane Bias tool, and JBI critical checklist in the selected studies are summarized in Table 2, Table 3 and Table 4. Based on the NOS, eight studies were rated as a total score of 7, one study scored 6, and the remaining four studies scored 8. Overall, the score of included studies was 7. The Cochrane bias tool assessed that almost all the domains for RCT were at low risk of bias. As per the JBI critical checklist, the quality of case reports in both reports were of good quality.

### 3.3. Dosing Strategy of TMP-SMX in Selected Studies

Table 1 shows the dosing regimen of TMP-SMX and their clinical outcomes. Of 13 studies, four reported that patients having AIDS received TMP-SMX for prophylaxis, four reported patients with rheumatoid arthritis, two documented non-HIV patients, two reported kidney transplant recipients, and one reported the patient with G6PD deficiency. Of 13 studies, five compared the low-dose regimen of TMP-SMX with a high-dose regimen [25,26,27,28,29]. These studies documented that low-dose TMP-SMX therapy must be considered as a first-line treatment option to prevent occurrence and recurrence of PCP and PCP-associated AEs. However, Ohmura and his colleagues reported that a low-dose treatment regimen of TMP-SMX with <10 mg/kg/day for TMP was as well-tolerated and effective as high-dose therapy [26]. Similarly, Schild et al. reported that the patients received an intermediate-dose TMP-SMX (TMP 10–15 mg/kg/day) but later on, 23% of patients switched to low-dose TMP-SMX due to various AEs and this regimen was deemed to be safe and effective in patients with various immune dysfunctions [12].

Low-dose treatment regimens usually reported fewer side effects compared to the conventional dosing regimens. However, a study reported that a in a total of 438 patients, the dose of TMP-SMX was reduced in 84 kidney transplant recipients for hyperkalemia, and 102 for leukopenia [30]. Another study reported side effects, such as hyperkalemia, with the use of TMP-SMX. Nakashima et al. reported that the total AEs rate was 58.3% in the low-dose group (TMP, 4–10 mg/kg/day; SMX 20–50 mg/kg/day), and in conventional-dose group (TMP, 10–20 mg/kg/day; SMX, 50–100 mg/kg/day), 72.4% of patients experienced AEs [29]. Similarly, another study reported that the mortality rates were lower in the low-dose group (19.5%) compared to the conventional-dose group (25.0%) [28]. A study documented that TMP-SMX was reported to cause hemolysis in patients with G6PD deficiency [32]. In both RCTs, no cases of PCP were documented up to weeks 24 and 52 [3,33].

## 4. Discussion

The main goal of this systematic review was to assess the current dosing strategy of TMP-SMZ to reduce the risk of PCP in patients especially those with various immunodeficiency syndromes. The impact of PCP on mortality and morbidity of the patients especially immunosuppressive patients is significant [36]. This review has revealed that low-dose TMP-SMX therapy should be the first-line treatment option, resulting in the reduction of mortality rate and PCP-associated AEs. The Cochrane meta-analysis reported that there was a decrease in the incidence of PCP up to 91% and decrease in mortality rate up to 83% when compared to the control group [9]. Although the TMP-SMX has been regarded as a first-line regimen for prophylaxis of PCP, it often has to be switched to second-line treatment such as dapsone, atovaquone, and atomized pentamidine due to TMP-SMX associated AEs and drug intolerance caused by neutropenia and glucose-6-phosphate dehydrogenase (G6PD) deficiency [9,10,36].

Over the past few years, various preventive treatment options are available for opportunistic infections in immunocompromised and transplanted patients [37]. Although highly recommended by the healthcare community, prophylactic drugs are included in the crucial immunosuppressive regimen, resulting in considerable economic burden for the immunocompromised patients [38]. Routine PCP prophylaxis is recommended for patients treated in hospitals where the prevalence of PCP is at least 3% to 5% among immunocompromised patients [12,39,40]. Moreover, the risk of PCP among transplant recipients is greater in 1–6 month post-transplantation period, during prolonged neutropenia, or in patients receiving steroids, antilymphocyte antibodies, or calcineurin inhibitors [41]. Other predisposing factors include concomitant cytomegalovirus, infections, number of graft rejection episodes, and low CD4+ count lymphocyte counts [38]. A study reported that 27% of the kidney transplant recipients receiving TMP-SMX prophylactically with other medications experienced AEs such as cytopenia [42]. Therefore, routine prophylaxis is mainly required during the early post-transplant month and after therapy of rejection episodes. PCP prophylaxis is highly recommended in HIV-positive patients who have CD4+ counts less than 200 cells/mm^3^ [43]. However, we failed to identify significant differences between the full-dose and low-dose regimen of TMP-SMX for the occurrence or recurrence of PCP. This strategy reduces economic burden of illness and enhances patients’ compliance to daily regimen plan.

Extrapolated from previous data, the first-line prophylactic agent is one single strength TMP-SMX tablet (TMP 80 mg/day; SMX 400 mg/day) or one double-strength (TMP 160 mg/day; SMX 800 mg/day) [44]. Alternative PCP prophylaxis includes one double-strength TMP-SMX tablet thrice per week, administration of dapsone either alone or in combination with leucovorin and pyrimethamine, atovaquone, or pentamidine sprays [44]. This practice may not provide desired clinical outcomes with the minimal risk of AEs. The double-strength seems to be effective while taken daily or thrice in a week, but daily administration of TMP-SMX prevents other post-transplant infectious diseases such as toxoplasmosis [45]. The standard dose of TMP-SMX was frequently used in an era before widespread solid organ transplantation and modern immunosuppressive treatment, which are now available for an ever-enlarging array of medical conditions [20,46].

Nowadays, patients with PCP differ from the patients that were treated 30–40 years ago [47]. The standard doses result in an absolute increase of 18% in the incidence of grade ≥3 AEs [46]. However, step-down therapy may be a worthy in exploring approaches to improve clinical outcomes [46]. The incidence of occurrence of PCP is the most frequent in patients with systemic rheumatoid arthritis disease taking steroids and immunosuppressive agents [26,48]. The risk factors for PCP in patients with rheumatoid disorders include age group (elderly > 65 years), use of glucocorticoids, and existing comorbidities [49]. In a study documenting the safety and efficacy of TMP-SMX given to patients with rheumatoid disorders taking high-dose steroids for PCP prophylaxis, the incidence of PCP and associated mortality rate was decreased after 1 year [35]. Moreover, another study showed that there were no proper guidelines on PCP prophylaxis in patients treated with immunosuppressive drugs, and high-dose steroids for immune-mediated dermatologic conditions [50]. Therefore, due to limited data on predictors and risk factors of PCP, the guidelines regarding PCP prophylaxis for various cohorts are also required in patients with rheumatological and other autoimmune dysfunctions [51].

This study does have several limitations. Firstly, the selected articles included patients from various countries, so the findings could have been affected by socio-demographic characteristics such as ethnicity, region, lifestyle, and environment. Secondly, the limited number of studies conducted after January 2000 forced us to include all studies that met inclusion criteria. Thirdly, although the quality of the majority of the included studies was good, due to small sample size, this may result in false-negative or false-positive findings.

## 5. Conclusions

These findings provided the current dosing strategy of TMP-SMX for the prevention and treatment of PCP. The low dose of TMP-SMX provides satisfactory outcomes while reducing the mortality rate and PCP-associated AEs. This strategy also reduces overall economic burden and enhances patients’ compliance to daily regimen plan. For some patient populations, such as PCP emerging on TMP-SMX prophylaxis in HIV-patients, it may still warrant to start prophylactic treatment regimen. Due to limited data available on the optimal dose of TMP-SMX, these findings would support the conduct of large-scale, prospective RCTs and cohort studies to provide guidelines regarding the dosing strategies for the PCP.

## Figures and Tables

**Table 1:** Dosing strategy of TMP-SMX in included studies.

Author and Year	Design	Sample Size	Characteristics of Patients	Dosing Regimen	Clinical Outcomes	Findings
Dao et al., 2014 [25]	Retrospective Cohort study	305	Patients with PCP infection	Group A received low-dose TMP-SMX regimen (TMP o15 mg/kg/day) while Group B received high-dose regimen (TMP 415 mg/kg/day)	In low-dose group, 32% of the patients were found to be within therapeutic range while in high-dose group, 22% of the patients were in therapeutic range	Furthers studies are required on large-scale to monitor plasma concentration of SMX and to evaluate the clinical outcomes.
Ohmura et al., 2018 [26]	Retrospective study	81	Patients with systemic rheumatoid diseases	Group A received low-dose SMX-TMP: ≤10 mg/kg/day; Group B received the intermediate dose, 10–15 mg/kg/day; Group C received high and conventional dose, 15–20 mg/kg/day for TMP dose.	The survival rate of Group A, B and C were 100%, 93.3%, and 96.7%, respectively.	Low-dose SMX/TMP treatment with ≤10 mg/kg/day for TMP was as safe and effective as high-dose regimen for occurrence and recurrence of PCP.
Yamashita et al., 2021 [27]	Retrospective study	81	Patients with HIV	Group A: standard-dose (≥6 SS (TMP-SMX 80 mg/400 mg tablets/week) Group B: low-dose groups (<6 SS tablets/week).	PCP was not developed in any patients during study period	Low-dose TMP-SMX is optimal treatment option to treat and prevent PCP
Schild et al., 2015 [13]	Observational Cohort study	104	Patients with PCPs in various immune dysfunctions	Patients received intermediate-dose TMP–SMX (TMP 10–15 mg/kg/day) and reduced to low-dose TMP–SMX (TMP 4–6 mg/kg/day) during therapy.	23% of patients were switched to low-dose TMP–SMX in step-down group compared to intermediate dose group	A step-down strategy to low-dose TMP–SMX also reported to be effective and safe
Kosaka et al., 2017 [28]	Retrospective cohort study	82	Patients with non-HIV-PCP	Group A received conventional dose of TMP (15 to 20 mg/kg), Group B received a low dose of TMP <15 mg/kg	The mortality rates were 25.0% in conventional-dose group and 19.5% in low-dose groups	The low-dose regimen is well tolerated and results in fewer adverse effects
Nakashima et al., 2018 [29]	Retrospective cohort study	24	Patients with non-HIV-PCP	Patients received low-dose TMP-SMX (TMP, 4e10 mg/kg/day; SMX, 20–50 mg/kg/day and conventional dose TMP-SMX (TMP, 10–20 mg/kg/day; SMX, 50–100 mg/kg/day) was used as reference	The total adverse reaction rate was 58.3% and 72.4% in low-dose group and conventional-dose group	Low-dose TMP-SMX may be considered as better treatment option for patients with non-HIV PCP
Prasad et al., 2019 [30]	Retrospective study	438	Kidney transplant recipients	SS dose of TMP-SMX OD, thrice daily and twice daily	The dose was reduced in 84 patients who experienced hyperkalemia and 102 patients who experienced leukopenia	TMP-SMX dose reduction is frequent in the first post-transplant year, but PCP does not occur
Rehman et al., 2021 [31]	Case report	01	Patient with CAP	-	Respiratory condition improved on day 9	Early diagnosis and management with TMP-SMX can lead to a better prognosis for patient
Lu et al., 2020 [32]	Case report	01	Patients with G6PD	TMP-SMZ (240/1200 mg) every 8 h, given IV. On day 16, PO (240/1200 mg) TID for 5 days	TMP-SMX reported to cause hemolysis in patients	Successfully treated with PCP with high dose of TMP-SMZ without any symptoms.
Park et al., 2021 [35]	Retrospective cohort study	1092	Patients with PCP and rheumatoid arthritis	one SS tablet of TMP-SMX (400/80 mg) per day for prophylaxis	TMP-SMX reduced 1 year PCP incidence and related mortality	TMP-SMX prophylaxis significantly decreased the incidence of the PCP with a favorable safety profile in a patient with RA taking steroids
Utsunomiya et al., 2017 [3]	RCT	183	Patients with systemic Rheumatoid diseases	SS group (SMX-TMP of 400/80 mg daily). HS group (200/40 mg/day) ES group (initiated with 40/8 mg/day, increasing to 200/40 mg/day)	No cases of PCP were reported up to week 24	The daily HS regimen is deemed to be first-line treatment option for the prophylaxis of PCP in patients with rheumatic disorders
Utsunomiya et al., 2020 [33]	RCT	183	Patients with rheumatoid diseases	SS group (SMX-TMP 400/80 mg/day), HS group (200/40 mg/day) ES group (initiating at 40/8 mg/day) and increasing to 200/40 mg/day)	PCP did not develop in any of the patients by week 52	SMX-TMP 200 mg/40 mg might provide a favourable benefit-risk balance in PCP prophylaxis.
Zamarlicha et al., 2015 [34]	Retrospective cohort study	88	Kidney transplant recipient	SMX-TMP dosed at 1 single-strength tablet thrice weekly	SMX-TMP therapy was discontinued in 10 patients while 11 patients received atovaquone.	A low-dose SMX-TMP regimen of 1 SS tablet thrice weekly is safe and effective.

HIV = Human immunodeficiency virus; CAP = Community-acquired pneumonia; IV = intravenous; RCT = Randomized controlled trial; PCP = *Pneumocystis jirovecii* pneumonia; TMP-SMX = Trimethoprim-Sulfamethoxazole; TID = Three times a day. SS = Single strength; HS = Half strength; and ES; Escalation strength.

**Table 2:** Quality assessment of cohort studies.

	Selection				Comparability	Outcomes		
Reference	Representative of Exposed Studies A	Selection of Non-Exposed B	Ascertainment of Exposure C	Demonstration of Outcome D	Comparability of Cohort Studies on Basis of Design E	Assessment of Outcomes F	Adequacy of Follow-up G	Quality Score
Dao et al., 2014 [25]	*	*	*	*	*	**	*	8
Ohmura et al., 2018 [26]	*	*	*	*	*	*	*	7
Yamashita et al., 2021 [27]	*	*	*	*	*	*	*	7
Schild et al., 2016 [13]	*	*	*	*	*	*	-	6
Kosaka et al., 2017 [28]	*	*	*	*	*	**	-	7
Nakashima et al., 2017 [29]	*	*	*	*	*	**	-	7
Prasad et al., 2019 [30]	*	*	*	*	*	**	*	8
Park et al., 2021 [35]	*	*	*	*	*	**	*	8
Zmarlicha et al., 2015 [34]	*	*	*	*	*	**	*	8

A: * = truly representative or somewhat representative of average in target population. B: * = Drawn from the same community. C: * = Secured record or structured review. D: * = Yes, = No. E: * = Study controls for age, gender, and other factors. F: * = Record linkage or blind assessment, ** = Both. G: * = follow-up of all subjects.

**Table 3:** Risk of bias assessment for randomized controlled trials.

Study	Random Sequence Generation	Allocation Concealment	Blinding of Participants and Personnel	Blinding of Outcome Assessment	Incomplete Outcome Data	Selective Reporting	Other Bias
Utsunomiya et al., 2017 [3]	Low risk	Low risk	Low risk	Low risk	Low risk	Low risk	Unclear
Utsunomiya et al., 2020 [33]	Low risk	Low risk	Low risk	Low risk	Unclear	Low risk	Unclear

**Table 4:** Quality assessment of case reports.

Questions *	Q1	Q2	Q3	Q4	Q5	Q6	Q7	Q8	Quality Rating
Rehman et al., 2021 [31]	Yes	Yes	Yes	Yes	No	Yes	Yes	Yes	Good
Lu et al., 2020 [32]	Yes	Yes	Yes	Yes	Yes	Yes	Yes	Yes	Good

* JBI critical checklist.

## Data Availability

Data is available within the article.
